# Measurement of synaptic density in Down syndrome using PET imaging: a pilot study

**DOI:** 10.1038/s41598-024-54669-7

**Published:** 2024-02-26

**Authors:** Alexandra DiFilippo, Erin Jonaitis, Renee Makuch, Brianna Gambetti, Victoria Fleming, Gilda Ennis, Todd Barnhart, Jonathan Engle, Barbara Bendlin, Sterling Johnson, Benjamin Handen, Sharon Krinsky-McHale, Sigan Hartley, Bradley Christian

**Affiliations:** 1 Madison School of Medicine and Public Health, University of Wisconsin, Madison, WI USA; 2 University of Wisconsin-Madison Waisman Center, Madison, WI USA; 3 Department of Psychiatry, University of Pittsburgh, Pittsburgh, PA USA; 4 New York State Institute for Basic Research in Developmental Disabilities, Staten Island, NY USA

**Keywords:** Brain imaging, Neurodevelopmental disorders, Molecular neuroscience

## Abstract

Down syndrome (DS) is the most prevalent genetic cause of intellectual disability, resulting from trisomy 21. Recently, positron emission tomography (PET) imaging has been used to image synapses *in vivo*. The motivation for this pilot study was to investigate whether synaptic density in low functioning adults with DS can be evaluated using the PET radiotracer [^11^C]UCB-J. Data were acquired from low functioning adults with DS (*n* = 4) and older neurotypical (NT) adults (*n* = 37). Motion during the scans required the use of a 10-minute acquisition window for the calculation of synaptic density using SUVR_50–60,CS_ which was determined to be a suitable approximation for specific binding in this analysis using dynamic data from the NT group. Of the regions analyzed a large effect was observed when comparing DS and NT hippocampus and cerebral cortex synaptic density as well as hippocampus and cerebellum volumes. In this pilot study, PET imaging of [^11^C]UCB-J was successfully completed and synaptic density measured in low functioning DS adults. This work provides the basis for studies where synaptic density may be compared between larger groups of NT adults and adults with DS who have varying degrees of baseline cognitive status.

## Introduction

Down syndrome (DS) is the most prevalent genetic cause of intellectual disability, occurring in one out of every 707 live births in the United States^1^ and resulting from the triplication of chromosome 21^2^. DS is characterized by widespread impairments in cognitive, motor, and language ability^3,4^, with IQs and functional ability ranging from the mild to profound level of intellectual disability^5,6^. Individuals with DS also have a 90% lifetime risk of Alzheimer’s disease (AD) due to the gene-dose overexpression of amyloid precursor protein, resulting in high prevalence of neuropathologic amyloid plaques^7,8^. Feasible and valid imaging biomarkers of neurobiology underlying both baseline cognitive impairments in people with DS and AD-related cognitive decline is of high priority to the field. However, the wide range in baseline cognitive ability in people with DS presents challenges for research studies. Indeed, leading research consortiums on biomarkers of AD in DS often exclude people with DS who have low baseline cognitive levels^9^ due to concerns about floor effects on cognitive tests and difficulties obtaining imaging scans. Yet, the identification of feasible and meaningful imaging biomarkers with lower functioning adults with DS is essentially for their inclusion in clinical research and therapeutic studies and to better understand how AD progression may differ based on baseline cognitive ability. Detecting early AD-associated neurodegeneration in the DS brain requires knowledge of brain structure and function prior to the onset of clinical AD.

*Pathophysiological differences have been identified in DS individuals relative to their NT peers most notably in the cerebral cortex, hippocampus, and cerebellum.* Most in vivo studies identifying these changes have used MRI showing smaller normalized hippocampus, cerebellum, and cerebral cortex volumes in both DS children^10–12^ and adults^13–17^. Using ex vivo analyses, reduced numbers of neurons were found in the cerebellum and cerebral cortex in DS^18–20^ compared to age-matched neurotypical adults. Shortened dendrites and dendrites presenting with either a paucity of spines or covered with an abnormally large number of spines was reported in the cerebral cortex^21–25^ and hippocampus^23,26^ of people with DS. Synaptic vesicle proteins synaptophysin and SNAP-25 levels were decreased in the cerebral cortex, cerebellum, and hippocampus^27^ whereas synaptojanin-1 was found to be increased in the cerebral cortex^28,29^. The limited data on substructural changes in the brain of people with DS suggest differences beyond the gross morphological or volumetric differences exist and should be further explored. *The analyses completed here will focus on the regions reported to show either gross or substructural changes in brain structure: the cerebellum, the hippocampus, and the cerebral cortex.* The use of PET imaging permits the *in vivo* analysis of synapses in a larger number of DS individuals as well as longitudinal information.

While differences in brain volume may be evaluated using MRI, PET can provide powerful *in vivo* substructural and functional information related neuronal development and degeneration. The PET radioligand [^11^C]UCB-J binds to presynaptic synaptic vesicle protein SV2A^30–32^, expressed ubiquitously in all synapse terminals^33^, with high specificity^32,34, 35^ and exhibits near complete blockade by levetiracetam^35^. Recently PET imaging studies using [^11^C]UCB-J have revealed pathophysiological changes in synaptic function in samples of NT adults with epilepsy^30,32^, Alzheimer’s disease^36–39^, schizophrenia^31,40, 41^, and Parkinson’s disease^42–44^. To our knowledge, the present study is the first investigation to evaluate the feasibility of measuring synaptic density with PET imaging of [^11^C]UCB-J in adults with DS. As previous studies of the DS brain have shown smaller hippocampus, cerebellum, and cerebral cortex volumes, these regions were chosen to examine synaptic density differences in this feasibility study. Along with characterizing synaptic density in the DS brain, there is a need for studies to identify differences in synaptic density between NT and DS populations such that the neurodegenerative effects of AD and potential regenerative effects of therapeutic treatments may be accurately measured in DS individuals.

## Results

### Participant enrollment and data acquisition

All four (100%) of the participants with DS were male, ranging in age from 27 to 51 years (*M* = 36.2 ± 9.2 years old). The average age-based IQ (Stanford Binet score) was 47.3 (range: 47-50) with an age equivalent of 4.16 years (range: < 2–6.2 years). To understand how sex might affect inference, an exploratory analysis was performed assessing the relationship between sex and [^11^C]UCB-J SUVR_50–60_ in the NT (*n* = 37, 78.4% female, 65.3 ± 5.2 years old) group. No such relationship was found, consistent with earlier studies^45,46^ [see Supplemental Materials 1].

**Figure 1:**
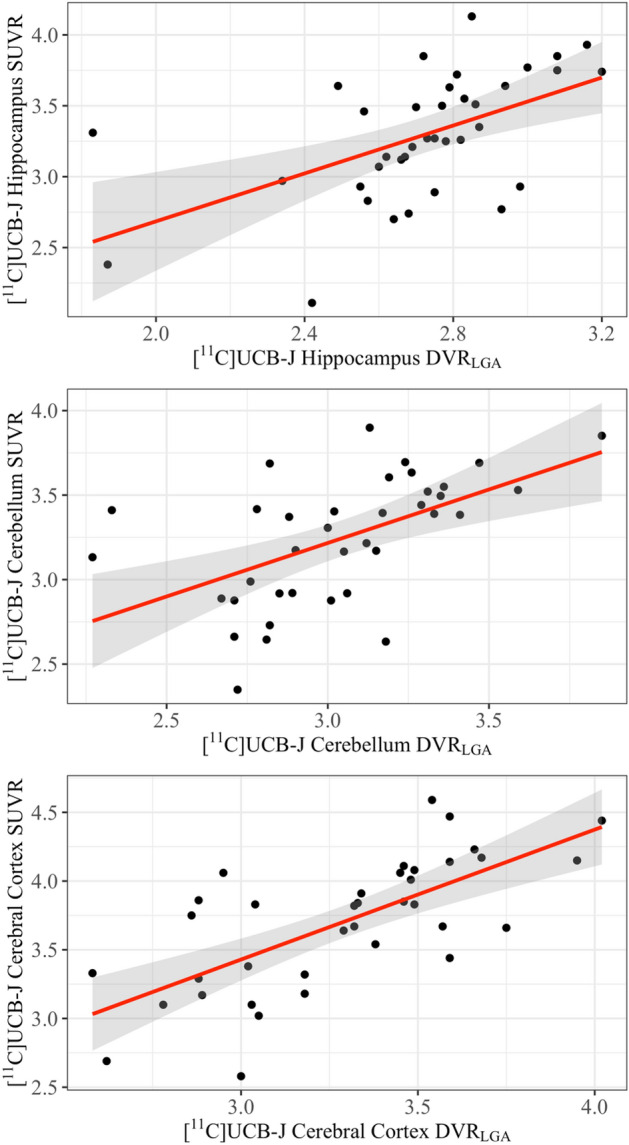
SUVR_50-60_ vs DVR_LGA_ in the hippocampus, cerebellum, and cerebral cortex of the neurotypical participants**.** A comparison of two measures of [^11^C]UCB-J specific binding, the distribution volume ratio (DVR_LGA_) and standardized uptake value ratio (SUVR_50-60_) in the hippocampus, cerebellum, and cerebral cortex of n = 37 neurotypical participants. Linear regression curves in red with corresponding 95% confidence interval in grey.

Significant motion during one of the DS [^11^C]UCB-J scans required the use of a 10-minute scan interval (50–60 min post-injection) for the SUVR calculation. The relationship between SUVR_50-60_ and distribution volume ratio (DVR_LGA_) was evaluated in the neurotypical group for validation of SUVR_50-60_ as a suitable estimate of [^11^C]UCB-J specific binding. A strong correlation was found between DVR_LGA_ and SUVR_50-60_ in the hippocampus (r = 0.65, p < 0.001), the cerebellum (r = 0.55, p < 0.001) and the cortex (r = 0.69, p < 0.001). To test for linearity, regression analysis was performed between SUVR_50-60_ and DVR_LGA_. The linear model (slope = 0.93, 95% CI [0.86, 1.00]; intercept = 0.51, 95% CI [0.30, 0.73]) fit the data well (hippocampus: R^2^ = 0.29; cerebellum: R^2^ = 0.30; cerebral cortex: R^2^ = 0.48; with all regions demonstrating significant relationships (p < 0.001)) [Fig. 1]. To check for heteroscedasticity, the model residuals were plotted against the fitted values. Heteroscedasticity was not observed and the linear relationship holds for all DVR values equally. The strong correlation and linearity of DVR_LGA_ and SUVR_50-60_ indicate that SUVR_50-60_ is a reasonable proxy for DVR_LGA_ in the neurotypical group.

### Global and regional synaptic density differences between DS and NT groups

**Figure 2:**
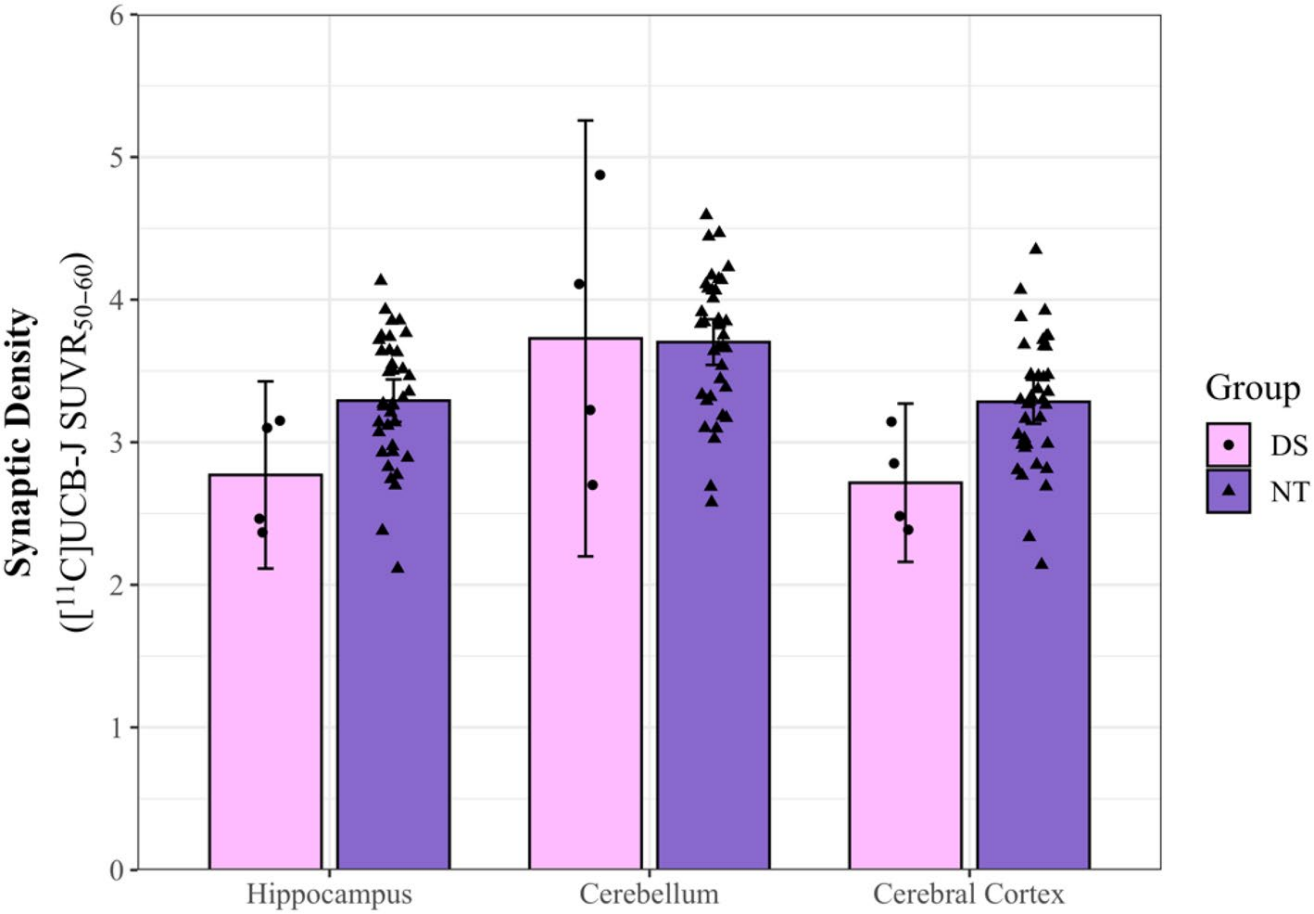
Comparison of synaptic density ([^11^C]UCB-J SUVR_50-60_) in neurotypical (NT) and Down syndrome (DS) groups. Data points represent [^11^C]UCB-J SUVR_50-60_ for each participant. Error bars show 95% confidence intervals.

The average DS hippocampus and cerebral cortex SUVR_50–60_ were lower than the NT group, with these differences showing a large effect size (Cohen’s *d* = 1.17, 1.25). This was still present in the hippocampus after PVC (*d* = 1.09) but not the cerebral cortex (*d* = − 0.29). There was no notable difference in cerebellum SUVR_50–60_ between groups [Table 1, Fig. 2].

**Table 1:** UCB-J ROI SUVR means, confidence intervals, and effect sizes.

Region	DS			NT			Cohen's d
	M	95% CI		M	95% CI		
		LL	UL		LL	UL	
Hippocampus	2.77	2.11	3.43	3.29	3.14	3.44	1.17
Cerebellum	3.73	2.20	5.26	3.70	3.54	3.86	− 0.05
Cerebral cortex	2.72	2.16	3.27	3.28	3.13	3.44	1.25

*CI* confidence interval, *LL* lower limit, *UL* upper limit.

### Global and regional brain volume differences between DS and NT groups

Regional brain volumes were corrected (vol_corr_) for intracranial volume (ICV), which showed no difference between groups (DS ICV = 1406.66 mL, 95% CI [1297.07, 1516.25]; NT ICV = 1408.29 mL, 95% CI [1361.62, 1454.96]).

**Figure 3:**
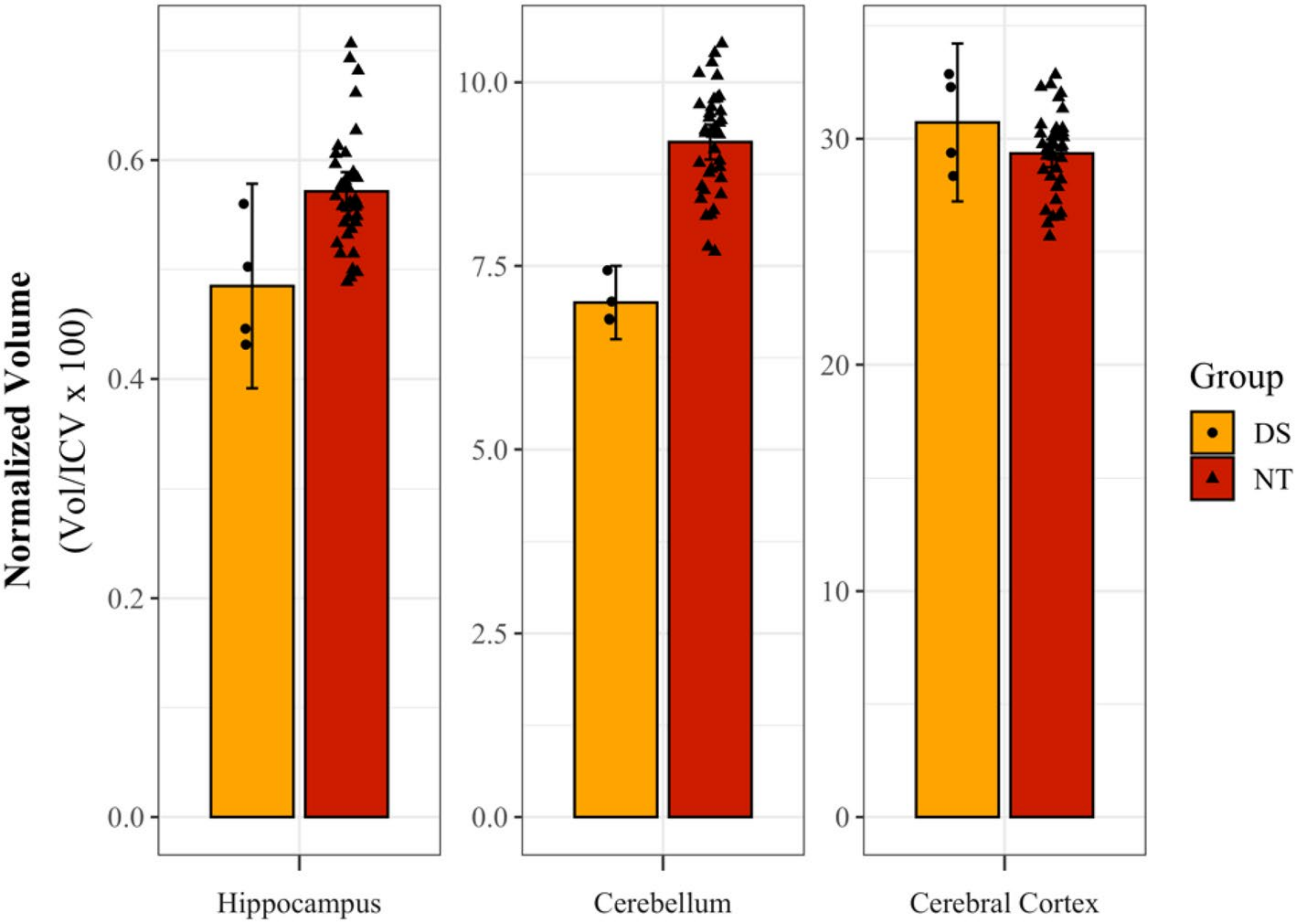
Comparison of brain region volume in neurotypical (NT) and Down syndrome (DS) groups. Normalized volume = 100* raw volume/intracranial volume. Data points represent normalized volumes for each participant with circles for DS and triangles for NT individuals. Error bars show 95% confidence intervals.

The average DS hippocampus and cerebellum volumes were lower than the NT group, with these differences showing a large effect (*d* = 1.62, 3.17). There was not a distinguishable difference in cerebral cortex volume between groups, and group averages were nearly identical [Table 2, Fig. 3].

**Table 2:** Normalized brain ROI volume means, confidence intervals, and effect sizes.

Region	DS			NT			Cohen's d
	M	95% CI		M	95% CI		
		LL	UL		LL	UL	
Hippocampus	0.49	0.39	0.58	0.57	0.55	0.59	1.62
Cerebellum	7.00	6.50	7.50	9.17	8.95	9.41	3.17
Cerebral cortex	30.72	27.23	34.22	29.28	28.67	29.89	− 0.74

*CI* confidence interval, *LL* lower limit, *UL* upper limit.

## Discussion

Our group has been actively involved in large scale PET imaging studies in the DS adult population to measure amyloid and tau burden related to AD in the brain^47–49^, highlighting the importance of obtaining AD-related neuroimaging biomarkers in individuals with DS and motivating their inclusion in investigations for understanding co-occurring illnesses across their lifespan (https://www.nia.nih.gov/research/abc-ds). These neuroimaging measures provide valuable information on the trajectory of AD neuropathologies which have been implicated in a cascade of events resulting in reduced synaptic density (via neurodegeneration). Concerns with experimental protocol compliance encountered by DS individuals with limited cognitive abilities have led to their exclusion from research studies and therapeutic trials, potentially diminishing their ability to benefit from advances in treatments and scientific findings. For PET radiotracer binding to serve as a reliable measure in the DS population, an imaging protocol must be feasible for all individuals and allow for the accurate calculation of radiotracer specific binding. The binding characteristics of the PET radiotracer [^11^C]UCB-J to SV2A have been carefully studied and used to validate its specific binding as an index of synaptic density^34^. The feasibility of evaluating synaptic density using [^11^C]UCB-J in DS adults with limited baseline cognitive abilities was evaluated in this study.

Due to motion during one of the DS PET scans, usable [^11^C]UCB-J emission data was limited to 2 frames, 50–60 minutes following radiotracer injection. Previously, only 30-minute windows were validated^50,51^ as sufficient for calculating synaptic density. When the NT cohort was used to examine the relationship between SUVR_50-60_ and DVR (0–70 min) a strong correlation with low variability was found. While the lengthier scanning procedure will improve the accuracy of the derived PET outcome, in situations where usable data is severely limited (e.g. due to motion) we see that a reliable metric of [^11^C]UCB-J specific binding may still be determined. In this feasibility study, biological sex was not matched across the DS and NT groups. However, this was not a concern as [^11^C]UCB-J binding has been studied in a large human cohort with findings suggesting sex-based differences in synaptic density are not seen in cortical nor subcortical regions^45,46^. While the DS group is thirty years younger than the NT group, based upon literature findings we do not expect a relationship between [^11^C]UCB-J binding and age^45,46^.

Following the imaging and kinetic analysis of [^11^C]UCB-J, the difference in SUVR_50–60_ between groups showed a large effect with average synaptic density in the hippocampus and the cerebral cortex in the DS group being lower than in the NT group. Following analysis of participant T1w-MRIs, previously reported findings of smaller hippocampus^14,52^ and cerebellum^10,13, 15–17,53^ volumes in the DS individuals relative to their NT peers were also seen here, but not the reductions in cerebral cortex volumes^10–12,17,54^. Due to the small number of DS participants used for these analyses, it will be important to see if these initial findings are replicated in larger samples. Additional data will also be needed to examine the relationship between synaptic density and the presence of AD-related neuropathology. As one of the DS participants is 51 years old, we cannot state that these results are not influenced by AD-related neuropathology due to amyloid accumulation beginning in individuals in their 40s^55^. As such, the data here may reflect baseline the neurodegenerative effects of AD.

Although preliminary, these findings are important given that previous investigations analyzing synaptic density and synaptic proteins in DS are limited to fetal and *ex-vivo* analyses. Such studies within the DS population have reported decreases in DS in synaptic density^56,57^ and specific synaptic protein expression^27,58^, along with no change^28^, or increased protein expression^28,29^. However, we hesitate to relate these as direct comparisons to the PET results due to differences in age of participants and methods of assay and sampling of brain tissue. Tissue-based assays of SV2A expression would provide valuable information to better understand the expression of [^11^C]UCB-J in DS.

There is a vast knowledge gap in our understanding of synaptic function accompanying Down syndrome spanning development between fetal and post mortem investigations. These findings demonstrate that there are not profound disruptions in synaptic formation and/or pruning, and likely, that the manifestation of intellectual disability is not the result of insufficient synaptic formation. However, these findings do not rule out the presence of smaller and more subtle deficiencies in synaptic density in brain regions or circuits related to cognitive domains that are affected in individuals with Down syndrome. These initial findings will serve to guide future study designs exploring in vivo neuronal and synaptic function in this medically underserved population. Further investigations with larger samples sizes will be critical not only confirm the findings of this pilot study but also to understand the relationship between neurodevelopment and neurodegeneration across the lifespan of individuals with DS.

## Methods

### Ethics approval and consent to participate

All procedures performed in studies involving human participants were in accordance with the ethical standards of the institutional and/or national research committee and with the 1964 Helsinki declaration and its later amendments or comparable ethical standards. Institutional Review Board (IRB) approval from the University of Wisconsin-Madison Health Sciences IRB and informed consent (and assent when appropriate) have been obtained from all study participants or their proxy/legally authorized representative (protocol: 2019-1464).

### Study participants and design

Participants with DS were recruited from the Alzheimer Biomarkers Consortium—Down Syndrome (ABC-DS) study^59^. This consortium was developed in part to identify factors contributing to AD-associated neurodegeneration and their effects on cognition in genetically confirmed DS. The ABC-DS study targets a standing cohort of 550 individuals with DS across 8 recruiting sites across the United States in addition to Cambridge University in the UK. For this neuroimaging pilot study, four participants with DS were recruited from a single site (University of Wisconsin-Madison) with the goal of demonstrating that DS individuals with limited cognitive ability (mental age ≤ 4 years or IQ < 30) could tolerate the PET and MRI scanning procedures. Prior to the scheduled study visit, participants and their caregivers were mailed a copy of the consent form (and assent form if applicable) and a copy of a video or DVD showing the MRI and PET scan procedures. Participants were asked to watch the video/DVD to better prepare themselves for the upcoming study visit. In some cases, the decision was made to cancel the scheduled visit once the individual observes what the procedures involve. Of the ten participants who were recruited for this study, six did not perform MRI and PET scanning due to issues including MRI weight limit, MRI-altering metal implants, and sensory limitations (i.e. auditory). As previously described, participants were administered a 2.5-hour battery of cognitive tests that assessed their overall intellectual level, visuospatial ability, motor planning and control, and executive functioning^59^ and completed an eye-tracking visual comparison test^60^. Caregivers/study partners reported on their medical and psychiatric history and completed informant reports of their behavior and everyday functioning. According to medical records, none of the participants had a history of seizure disorder. Blood samples were taken for apolipoprotein (APOE) genotyping and cytogenetic analysis. Because the total enrollment was only four participants, we elected not to balance for sex.

Data from NT participants was obtained from an ongoing study investigating AD-related synaptic loss (SYNAPSE) conducted at the University of Wisconsin-Madison affiliated with the Wisconsin Alzheimer’s Disease Research Center (ADRC) and the Wisconsin Research for Alzheimer’s Prevention (WRAP) cohorts that included [^11^C]UCB-J PET imaging. Participants in the SYNAPSE study underwent comprehensive clinical and cognitive evaluation to determine cognitive status according to NINDS/ADRDA criteria, and status was confirmed by a multidisciplinary consensus diagnostic panel. PET imaging with [^11^C]PiB and [^18^F]MK-6240 was completed to quantify beta amyloid plaque and neurofibrillary tau tangle burden, respectively. Participants selected for the present analysis did not have any evidence of cognitive impairment, significant global amyloid and neurofibrillary tau burden, or atrophy.

### Imaging procedures

*[*^*11*^*C]UCB-J Radiochemistry* Synthesis of [^11^C]UCB-J was based on previously described methods^35,61,62^ with modifications detailed here. To prepare the hydrolyzed precursor, a vial containing 1.8 ± 0.2 mg trifluoroborate precursor, 400 μL MeOH, and 200 μL 1 N HCl is stirred at 55° C for 15 minutes and then dried under argon. [^11^C]MeI (400–800 mCi at end of bombardment) is bubbled into a chilled 5 mL v-vial containing 222 μL DMF, 0.9 ± 0.2 mg Pd_2_(dba)_3_, 0.8 ± 0.2 mg P(o-tol)_3_, and 28 μL 0.215M K_2_CO_3_. The hydrolyzed precursor in 200 μL DMF is added to the reaction vessel and the solution stirred at 130 °C for 5 minutes. After the reaction vial is cooled, 1.6 mL 1 N HCl is added and the solution filtered prior to injection onto a semiprep HPLC system. The [^11^C]UCB-J fraction is collected and diluted in 50 mL sterile water. The diluted fraction is trapped on a Waters Sep-Pak tC18 cartridge which is rinsed with 10 mL 0.001 N HCl. The product is eluted with 1 mL EtOH and 10 mL saline with 20 μL 8.4% sodium bicarbonate and passed through a sterile filter. The final formulated dose had a specific activity > 20 mCi/nmol with a radiochemical purity > 99%. *PET Acquisition* [^11^C]UCB-J PET imaging was performed on a Siemens ECAT HR+ PET or a Siemens Biograph Horizon PET/CT scanner, with both scanners having comparable spatial resolution (~ 6 mm as acquired). Participants received an intravenous injection of 14.4 ± 2.5 mCi [^11^C]UCB-J. NT participants were scanned for 70 minutes initiated with injection for distribution value ratio calculation. Originally as part of the study design the DS participants were to complete a 60-minute acquisition for the calculation of the distribution volume ratio. The first DS participant was scanned using a full dynamic acquisition. However, an abbreviated acquisition was implemented for subsequent participants to minimize the scanning duration. The abbreviated scan included a 40- or 50-minute uptake period before being positioned in the PET scanner for a 30 min. acquisition. While this scan start time is different than the 60–90 minute window characterized by Naganawa et al. (2021), an earlier start time was chosen to ensure an overlap in acquisition times between all scans. *MRI Acquisition* Structural T1-weighted (MPRAGE) MRI scans were obtained using 3T GE Signa 750 MRI scanner to guide processing of PET scans and region of interest (ROI) identification.

### Data analysis

PET data was reconstructed using filtered back-projection and corrected for photon attenuation, deadtime, normalization, scatter, and radioactive decay. Using automated methods, PET-MRI registration was performed with SPM12. The T1-weighted MRI scans were segmented and parcellated using the FreeSurfer software version 6^63^. The FreeSurfer grey matter segmentation of the Desikan-Killiany atlas regions^64,65^ was used to identify the hippocampus, cerebellum, and cerebral cortex ROIs and their volumes. The hippocampus was not originally included as a region of interest due to its small size and the small DS group size, but upon further research of previous studies there was increased motivation to include it as an ROI due to previously reported volume decreases. [^11^C]UCB-J standardized uptake value ratio (SUVR) was used as an index of synaptic density with the centrum semiovale as the reference region. The centrum semiovale ROI was identified using MRI-guided manual parcellation. Previous studies have validated^50,51^ an abbreviated 30-minute scan interval as sufficient for calculating synaptic density as the SUVR approximation correlated well with the more quantitatively accurate index of distribution volume ratio (DVR), calculated using the full 70-minute PET data acquisition. For this study, usable PET emission data was restricted to 10 minutes (50–60 minutes post-injection) due to motion during one of the participant’s scans. To determine whether synaptic density could be accurately calculated despite the reduced scanning duration, an analysis was performed in the NT group to characterize the relationship between the 10-minute SUVR_50-60_ and the DVR outcome. Bilateral ROI SUVR and volume measures were calculated using the voxel-weighted averages from the individual cortical and subcortical regions.

### Statistical methods

All analyses were performed using R Statistical Software (v4.3.0; R Core Team 2023). Pearson’s correlation and the coefficient of determination were used to evaluate the use of SUVR as a proxy measure for DVR. As this study is underpowered to draw conclusions on differences in ROI UCB-J SUVR and volume between DS and NT population, results are restricted to descriptions of averages, confidence intervals, and effect sizes (Cohen’s d). Effect sizes were also evaluated following partial volume correction (PVC) using a region-based voxelwise method.

### Supplementary Information

Supplementary Information 1.

## Data Availability

Imaging data used and/or analyzed during the current study are available from the corresponding author on reasonable request.

## References

[1] Mai CT, Isenburg JL, Canfield MA (2019). National population-based estimates for major birth defects, 2010–2014. Birth Defects Res..

[2] Lejeune J, Gautier M, Turpin R (1958). Tudesdes chromosomes somatiques de neuf enfantsmongolieus [Studies of somatic chromo-somes of nine mongoloid children]. Com-petes Renders de l’Academie des Sciences Serie III.

[3] Lott IT, Dierssen M (2010). Cognitive deficits and associated neurological complications in individuals with Down’s syndrome. Lancet Neurol..

[4] Risgaard KA, Sorci IA, Mohan S, Bhattacharyya A (2022). Meta-analysis of Down syndrome cortical development reveals underdeveloped state of the science. Front. Cell Neurosci..

[5] Hamburg S, Lowe B, Startin CM (2019). Assessing general cognitive and adaptive abilities in adults with Down syndrome: A systematic review. J. Neurodev. Disord..

[6] Chapman RS, Hesketh LJ (2000). Behavioral phenotype of individuals with Down syndrome. Ment. Retard. Dev. Disabil. Res. Rev..

[7] Lott IT, Head E (2001). Down syndrome and Alzheimer’s disease: A link between development and aging. Mental Retard. Dev. Disabil. Res. Rev..

[8] Head E, Powell D, Gold B, Schmitt F (2014). Alzheimer’s disease in Down syndrome. Eur. J. Neurodegenerat. Dis..

[9] Handen BL, Lott IT, Christian BT (2020). The Alzheimer’s biomarker Consortium-Down syndrome: Rationale and methodology. Alzheimer Dement. Diagn. Assess. Dis. Monit..

[10] Pinter J, Eliez S, Schmitt JE, Capone GT, Reiss AL (2001). Neuroanatomy of Down’s syndrome : A high-resolution MRI study. Am. J. Psychiatry.

[11] Kates WR, Folley BS, Lanham DC, Capone GT (2000). Cerebral growth in fragile X syndrome: Review and comparison with Down syndrome. Microscopy Res. Techn..

[12] Carter JC, George T (2008). Neuroanatomic correlates of autism and stereotypy in children with Down syndrome. NeuroReport.

[13] Raz N, Torres IJ, Briggs SD (1995). Selective neuroanatomic abnormalities in down’s syndrome and their cognitive correlates: Evidence from MRI morphometry. Neurology.

[14] Aylward EH, Li Q, Honeycutt NA (1999). MRI volumes of the hippocampus and amygdala in adults with Down’s syndrome with and without dementia. Am. J. Psychiatry.

[15] White NS, Alkire MT, Haier RJ (2003). A voxel-based morphometric study of nondemented adults with Down Syndrome. Neuroimage.

[16] Aylward EH, Habbak R, Warren AC (1997). Cerebellar volume in adults with Down syndrome. Arch. Neurol..

[17] Weis S, Weber G, Neuhold A, Rett A (1991). Down syndrome: MR quantification of brain structures and comparison with normal control subjects. Am. J. Neuroradiol..

[18] Coyle JT, Oster-Granite ML, Gearhart JD (1986). The neurobiologie consequences of down syndrome. Brain Res Bull..

[19] Contestabile A, Fila T, Ceccarelli C (2007). Cell cycle alteration and decreased cell proliferation in the hippocampal dentate gyrus and in the neocortical germinal matrix of fetuses with down syndrome and in Ts65Dn mice. Hippocampus.

[20] Larsen KB, Laursen H, Græm N, Samuelsen GB, Bogdanovic N, Pakkenberg B (2008). Reduced cell number in the neocortical part of the human fetal brain in Down syndrome. Ann. Anat..

[21] Takashima S, Ieshima A, Nakamura H, Becker LE (1989). Dendrites, dementia and the down syndrome. Brain Dev..

[22] Marin-Padilla M (1976). Pyramidal cell abnormalities in the motor cortex of a child with Down’s syndrome. A Golgi study. J. Comp. Neurol..

[23] Sarnat HB, Flores-Sarnat L (2021). Excitatory/inhibitory synaptic ratios in polymicrogyria and down syndrome help explain epileptogenesis in malformations. Pediatr. Neurol..

[24] Marin-Padilla M (1972). Structural abnormalities of the cerebral cortex in human chromosomal aberrations: A Golgi study. Brain Res..

[25] Purpura DP (1975). Normal and Aberrant Neuronal Development in the Cerebral Cortex of Human Fetus and Young Infant.

[26] Suetsugu M, Mehraein P (1980). Spine distribution along the apical dendrites of the pyramidal neurons in Down’s syndrome—A quantitative Golgi study. Acta Neuropathol..

[27] Downes EC, Robson J, Grailly E (2008). Loss of synaptophysin and synaptosomal-associated protein 25-kDa (SNAP-25) in elderly Down syndrome individuals. Neuropathol. Appl. Neurobiol..

[28] Martin SB, Dowling ALS, Lianekhammy J (2014). Synaptophysin and synaptojanin-1 in down syndrome are differentially affected by Alzheimer’s disease. J. Alzheimer Dis..

[29] Arai Y, Ijuin T, Takenawa T, Becker LE, Takashima S (2002). Excessive expression of synaptojanin in brains with Down syndrome. Brain Dev..

[30] Finnema SJ, Toyonaga T, Detyniecki K (2020). Reduced synaptic vesicle protein 2A binding in temporal lobe epilepsy: A [11C]UCB-J positron emission tomography study. Epilepsia.

[31] Onwordi EC, Whitehurst T, Mansur A (2021). The relationship between synaptic density marker SV2A, glutamate and N-acetyl aspartate levels in healthy volunteers and schizophrenia: A multimodal PET and magnetic resonance spectroscopy brain imaging study. Transl. Psychiatry.

[32] Finnema SJ, Nabulsi NB, Mercier J (2017). Kinetic evaluation and test-retest reproducibility of [11 C]UCB-J, a novel radioligand for positron emission tomography imaging of synaptic vesicle glycoprotein 2A in humans. J. Cereb. Blood Flow Metab..

[33] Stout KA, Dunn AR, Hoffman C, Miller GW (2019). The Synaptic vesicle glycoprotein 2: Structure, function, and disease relevance. ACS Chem. Neurosci..

[34] Finnema SJ, Nabulsi NB, Eid T (2016). Imaging synaptic density in the living human brain. Neurology.

[35] Nabulsi NB, Mercier J, Holden D (2016). Synthesis and preclinical evaluation of 11C-UCB-J as a PET tracer for imaging the synaptic vesicle glycoprotein 2A in the brain. J. Nucl. Med..

[36] Mecca AP, Chen MK, O’Dell RS (2022). Association of entorhinal cortical tau deposition and hippocampal synaptic density in older individuals with normal cognition and early Alzheimer’s disease. Neurobiol. Aging.

[37] Vanderlinden G, Ceccarini J, Casteele TV (2022). Spatial decrease of synaptic density in amnestic mild cognitive impairment follows the tau build-up pattern. Mol. Psychiatry.

[38] Chen MK, Mecca AP, Naganawa M (2021). Comparison of [11C]UCB-J and [18F]FDG PET in Alzheimer’s disease: A tracer kinetic modeling study. J. Cereb. Blood Flow Metabol..

[39] O’Dell RS, Mecca AP, Chen MK (2021). Association of Aβ deposition and regional synaptic density in early Alzheimer’s disease: A PET imaging study with [11C]UCB-J. Alzheimers Res. Ther..

[40] Radhakrishnan R, Skosnik PD, Ranganathan M (2021). In vivo evidence of lower synaptic vesicle density in schizophrenia. Mol. Psychiatry.

[41] Onwordi EC, Halff EF, Whitehurst T (2020). Synaptic density marker SV2A is reduced in schizophrenia patients and unaffected by antipsychotics in rats. Nat. Commun..

[42] Delva A, van Laere K, Vandenberghe W (2022). Longitudinal positron emission tomography imaging of presynaptic terminals in early Parkinson’s disease. Published Online.

[43] Matuskey D, Tinaz S, Wilcox KC (2020). Synaptic changes in Parkinson disease assessed with in vivo imaging. Ann. Neurol..

[44] Andersen KB, Hansen AK, Damholdt MF (2021). Reduced synaptic density in patients with Lewy body dementia: An [11C]UCB-J PET Imaging study. Mov. Disord..

[45] Michiels L, Delva A, van Aalst J (2021). Synaptic density in healthy human aging is not influenced by age or sex: A 11C-UCB-J PET study. Neuroimage.

[46] Naganawa M, Gallezot JD, Finnema SJ (2021). Simplified quantification of 11C-UCB-J PET evaluated in a large human cohort. J. Nucl. Med..

[47] Lao PJ, Handen BL, Betthauser TJ (2019). Imaging neurodegeneration in Down syndrome: Brain templates for amyloid burden and tissue segmentation. Brain Imaging Behav..

[48] Zammit MD, Laymon CM, Tudorascu DL (2020). Patterns of glucose hypometabolism in Down syndrome resemble sporadic Alzheimer’s disease except for the putamen. Alzheimer Dement. Diagn. Assess. Dis. Monit..

[49] Hartley SL, Handen BL, Tudorascu D (2022). Role of tau deposition in early cognitive decline in Down syndrome. Alzheimer Dement. Diagn. Assess. Dis. Monit..

[50] Naganawa M, Gallezot JD, Finnema SJ, Matuskey D (2020). Simplified quantification of 11 C-UCB-J PET evaluated in a large human cohort. J. Nucl. Med..

[51] Mertens N, Maguire RP, Serdons K (2019). Validation of parametric methods for [11 C]UCB-J PET imaging using subcortical white matter as reference tissue. Mol. Imaging Biol. Published Online.

[52] Pinter JD, Brown WE, Eliez S, Schmitt JE, Capone GT, Reiss AL (2001). Amygdala and hippocampal volumes in children with Down syndrome: A high-resolution MRI study. Neurology.

[53] Patkee PA, Baburamani AA, Kyriakopoulou V (2019). Early alterations in cortical and cerebellar regional brain growth in Down Syndrome: An in vivo fetal and neonatal MRI assessment. Neuroimage Clin..

[54] Lee SJC, Nam E, Lee HJ, Savelieff MG, Lim MH (2017). Towards an understanding of amyloid-β oligomers: Characterization, toxicity mechanisms, and inhibitors. Chem. Soc. Rev..

[55] Zammit MD, Tudorascu DL, Laymon CM (2020). PET measurement of longitudinal amyloid load identifies the earliest stages of amyloid-beta accumulation during Alzheimer’s disease progression in Down syndrome. Neuroimage.

[56] Weick JP, Held DL, Bonadurer GF (2013). Deficits in human trisomy 21 iPSCs and neurons. Proc. Natl. Acad. Sci. U. S. A..

[57] Hibaoui Y, Grad I, Letourneau A (2014). Modelling and rescuing neurodevelopmental defect of Down syndrome using induced pluripotent stem cells from monozygotic twins discordant for trisomy 21. EMBO Mol. Med..

[58] Lauterborn JC, Cox CD, Chan SW, Vanderklish PW, Lynch G, Gall CM (2020). Synaptic actin stabilization protein loss in Down syndrome and Alzheimer disease. Brain Pathol..

[59] Handen BL, Lott IT, Christian BT (2020). The Alzheimer’s biomarker consortium-down syndrome: Rationale and methodology. Alzheimer Dement. Diagn. Assess. Dis. Monit..

[60] Bott NT, Lange A, Rentz D, Buffalo E, Clopton P, Zola S (2017). Web camera based eye tracking to assess visual memory on a visual paired comparison task. Front. Neurosci..

[61] Xin Y, DiFilippo A, Murali D, et al. Improved synthesis of [11C]UCB-J for PET Imaging of SV2A. *J. Nucl. Med*. 63, (2022).

[62] DiFilippo A, Murali D, Ellison P, Barnhart T, Engle J, Christian B. Improved synthesis of [11C]UCB-J for PET imaging of synaptic density. *J. Nucl. Med*. 60, (2019).

[63] Fischl B (2012). FreeSurfer. Neuroimage.

[64] Desikan RS, Ségonne F, Fischl B (2006). An automated labeling system for subdividing the human cerebral cortex on MRI scans into gyral based regions of interest. Neuroimage.

[65] Klein A, Tourville J (2012). 101 labeled brain images and a consistent human cortical labeling protocol. Front. Neurosci..

