# Supplementary material for: Measurement of synaptic density in Down syndrome using PET imaging: a pilot study
Source: Sci Rep. 2024 Feb 26;14:4676. doi: 10.1038/s41598-024-54669-7 (PMC10897336; doi:10.1038/s41598-024-54669-7)
Supplement: Supplementary file 1 — Supplementary Information 1. [file 41598_2024_54669_MOESM1_ESM.pdf]

Supplemental Material 1

Analysis of sex-based differences in neurotypical SUVRs by ROI.

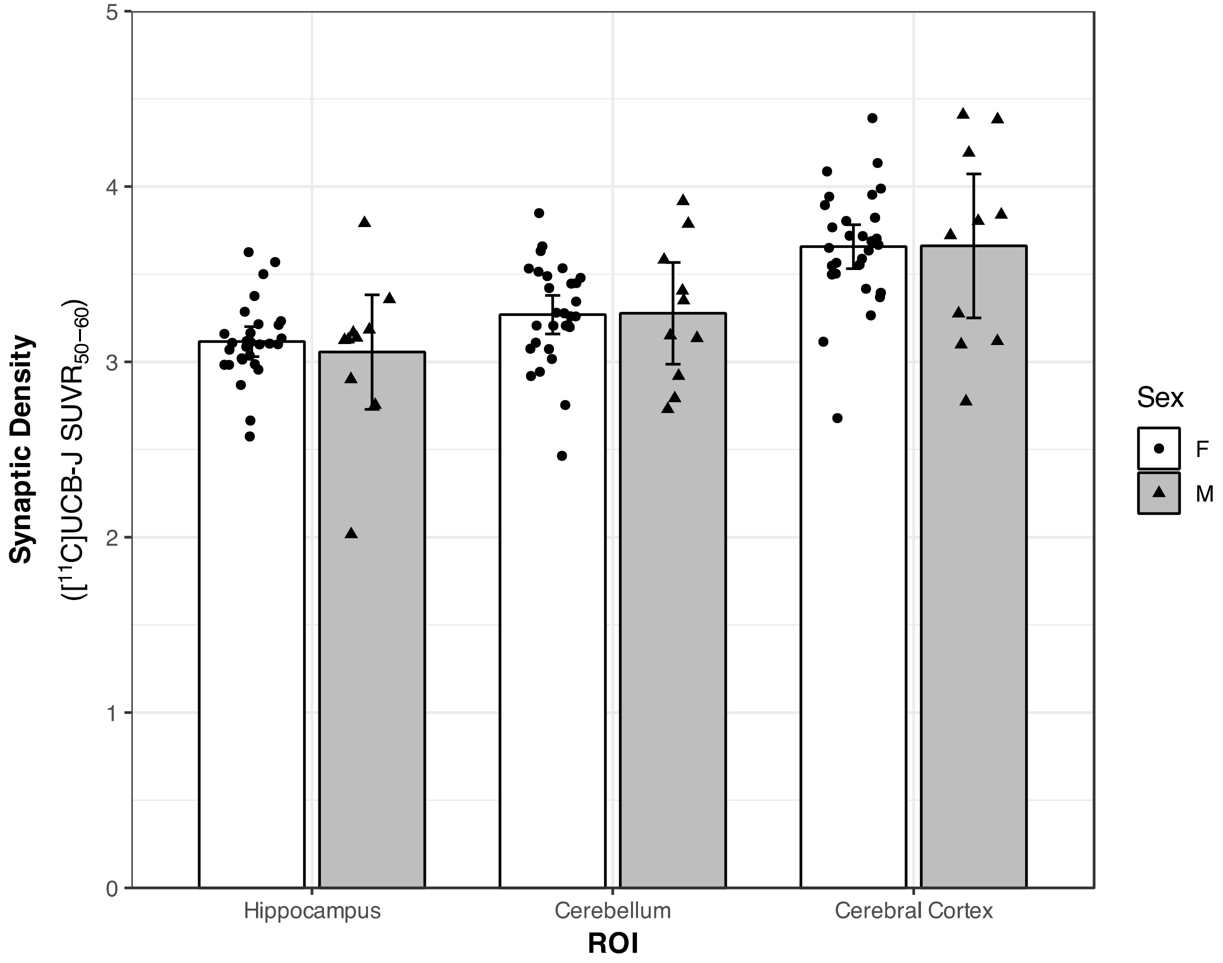

Note Error bars show 95% confidence interval.

Sex-based synaptic density differences in NT group

As the Down syndrome group is entirely male, [^{11}C]UCB-J SUVR was evaluated in the hippocampus, cerebellum, and cerebral cortex of the NT group for sex-based differences. In the neurotypical group (29 females, 9 males) no statistically significant sex-based differences in SUVR was seen in any ROI (see Figure 1) (Student's t-test $p > 0.05$).
